# Cross sectional study of factors associated to self-reported blood-borne infections among drug users

**DOI:** 10.1186/s12889-015-2442-6

**Published:** 2015-11-13

**Authors:** Juliana Reyes-Urueña, M. Teresa Brugal, Xavier Majo, Antonia Domingo-Salvany, Joan A. Caylà

**Affiliations:** Department of Paediatrics, Gynaecology and Preventive Medicine, Universidad Autónoma de Barcelona, Barcelona, Spain; Drug Use Epidemiology Research Group, IMIM-Hospital del Mar Medical Research Institute, Dr Aiguader 88, 08003 Barcelona, Spain; Teaching Unit of Preventive Medicine and Public Health, PSMAR-UPF-ASPB, Barcelona, Spain; Public Health Agency of Barcelona, Barcelona, Spain; Institute of Biomedical Research Sant Pau (IIB Sant Pau), Barcelona, Spain; Department of Health Government of Catalonia, Barcelona, Spain; CIBER in Epidemiology and Public Health (CIBERESP), Barcelona, Spain

**Keywords:** Drug users, Illicit drugs, HIV, Hepatitis, Spain, Risk Factors

## Abstract

**Background:**

The study’s aim was to estimate the self-reported prevalence of Human Immunodeficiency Virus (HIV) and Hepatitis C Virus (HCV), and to describe their associated risk factors in a population of users of illicit drugs recruited in Catalonia- Spain, during 2012.

**Methods:**

Cross-sectional study. People with illicit drugs use were selected in three different types of healthcare centres. The questionnaire was a piloted, structured ad hoc instrument. An analysis was made to identify factors associated to self-reported HCV, HIV and co-infection. Correlates of reported infections were determined using univariate and multivariate Poisson regression (with robust variance).

**Results:**

Among 512 participants, 39.65 % self-reported positive serostatus for HCV and 14.84 % for HIV, co-infection was reported by 13.48 %. Among the 224 injecting drug users (IDUs), 187 (83.48 %), 68 (30.36 %) and 66 (29.46 %) reported being positive for HCV, HIV and co-infection, respectively. A higher proportion of HIV-infected cases was observed among women, (18.33 % vs. 13.78 % in men). Prevalence of HCV, HIV and co-infection were higher among participants with early onset of drug consumption, long periods of drug injection or who were unemployed. A positive serostatus was self-reported by 21(7.34 %) participants who did not report any injection; among them 16 and eight, reported being positive for HCV and HIV, respectively; three reported co-infection. Only two people declared exchanging sex for money. For those that reported a negative test, the median time since the last HIV test was 11.41 months (inter-quartile range (IQR) 4–12) and for the HCV test was 4.5 months (IQR 2–7).

**Conclusions:**

Among drug users in Catalonia, HIV, HCV and co-infection prevalence are still a big issue especially among IDUs. Women and drug users who have never injected drugs are groups with a significant risk of infection; this might be related to their high-risk behaviours and to being unaware of their serological status.

## Background

Drug dependence is a complex chronic condition, which is often related to health problems, such as the risk of acquisition of blood-borne pathogens. People who use drugs, especially by injection, have been among the first and largest transmission group for new human immunodeficiency virus (HIV) infections [1], and are still a key transmission group for hepatitis C virus (HCV) [2]. These infections are among the most costly consequences of illicit drug use, having a high impact on individuals and on healthcare systems [3].

Epidemiological studies have described different transmission risk factors for HIV/HCV among drug users. Injecting drugs is the leading source of infection, and among non-injecting users it is sexual contact [4]. Among injecting drug users (IDUs), HIV has a global prevalence of approximately 11.5 % and HCV of 51.0 % [5], mainly as a result of sharing contaminated syringes and other injecting equipment [6, 7]. Unprotected sex is one of the most important risk factors for HIV in drug users who have never injected drugs [4], with an increased risk of infection by HCV among women and men who have sex with men who are co-infected with HIV or other sexually transmitted diseases [8]. Other sex-related factors associated with the increased risk of blood-borne infections (BBI) include a history of a sexually transmitted disease [9], sex with an IDU partner [10], exchanging sex for money or drugs [11] and number of sex partners [12].

Data from surveillance systems and cohort studies suggest that prevalence of both infections are high among IDUs. HCV is five times more prevalent than HIV, and has remained unchanged even in communities where the rate of HIV infections has fallen [13]. In Spain, HIV prevalence in IDUs seems to be decreasing, although it is still high compared with other European countries [14]. In contrast prevalence of HCV among IDUs has remained stable or increased slightly in recent years [3]. In Catalonia, a Spanish Autonomous Community, one study showed that during 2006, prevalence of HIV and HCV among IDUs were 58.1 and 80.1 %, respectively [15], with 50.3 % of co-infection.

In view of the above factors, drug users constitute a vulnerable group needing continual surveillance, especially because they constitute a key group in which to focus preventive interventions that can reduce transmission of BBI. The objective of this study was to estimate the prevalence of self-reported HIV and HCV, and to describe factors associated with them, in a large sample of consumers of illicit drugs recruited in Catalonia- Spain, during 2012.

## Methods

### Study design and population

Cross-sectional study in Catalonia, Spain, where participants were selected from three different drug healthcare settings: 1) Substance abuse outpatient treatment centres (OTC), 2) Harm Reduction facilities (HRF) and 3) Therapeutic communities (TC). Sampling was planned to achieve good geographical coverage. Each centre was assigned a number of participants to be recruited, according to their activity, over-sampling the smallest, especially in HRF. In the OTC this was done only for those having more than 45 annual patients; and in each OTC a convenience sample was selected taking into account whether first treatment and time in treatment. In the HRF, individuals were paid 10€ for their participation. All participants who agreed to participate signed a consent form. The study was approved by the IMIM-Hospital del Mar Medical Research Institute ethical committee. Field-work was conducted from April to June 2012. Serologic testing was not used; therefore infection prevalence calculated in the analysis are based on self-reported data.

## Questionnaire

The questionnaire was a piloted, structured ad hoc instrument. The questionnaire was anonymised and included questions related to socio-demographic characteristics (age, sex, country of birth, marital status, educational level and employment status), patterns of drug consumption (drugs, periods of consumption and whether or not injecting), prison history (ever been convicted of a crime or imprisoned) and self-reported HIV and HCV serological status. Information on HCV and HIV serological status was retrieved from questions asking whether they had ever had a blood test for HIV/HCV (one at a time) and, if so, when was the last test and whether a positive result was ever given. Injecting drug users were defined as individuals reporting any drug injection at least once in their lifetime.

## Statistical analysis

An analysis was made to identify factors associated to self-reported HIV, HCV and co-infection. Self-reported co-infected individuals were included in the total number of infected individuals for self-reported HIV and self-reported HCV when analysed separately. Only subjects reporting positive serology were considered as such and were compared to subjects either negative or with unknown serostatus. The descriptive analysis included socio-demographic characteristics, pattern of drug consumption and prison history. Comparisons were made to provide insight into which factors were linked to self-reported blood borne infections. Correlates were determined using a Poisson regression with robust variance. Poisson regression was used instead of logistic regression because of the high prevalence of infections and because it allows for direct estimation of self-reported prevalence ratios (PRs), along with 95 % confidence intervals [CIs]. The chi-square test for categorical variables was used to compare self-reported BBI differences among IDUs and drug users who have never injected drugs.

Multivariate models were constructed to identify independent correlates of self-reported infection for each HIV/HCV self-reported infection and co-infection. The covariates included into the model were: age, sex, country of birth, current marital status, educational level, current employment status, injecting drug user, and prison history. Models included covariates determined to be statistically important on the basis of associations with self-reported infection in the univariate analysis (*p* < 0.20) and only the statistically significant ones were retained. The level of significance was set at *p* value < 0.05. Data analysis was performed using PASW Statistics for Windows, version 18.0.

## Results

Forty seven centres were included for the sampling of participants (22 OTC, 13 HRF and 12 TC). A total of 558 individuals with illegal drugs use were approached for the study: 362 in OTC, 99 in HRF, and 97 in TC. Forty-two people refused to participate, due to: lack of time (45 %), were not interested (19 %) and unknown reasons (31 %). In addition, four questionnaires were excluded because they were incomplete (two of them didn’t report any information related to their serostatus).

A total of 512 individuals were studied, with a median age of 39 years, 76.56 % were male, 57.81 % had used heroin and 93.42 % had used cocaine. Overall, 210 subjects (41.02 %) self-reported being positive either for HIV and/or HCV; the prevalence of HCV was 39.65 %, for HIV it was 14.84 %, co-infection being reported by 13.48 % subjects. For all proportions, the denominator includes 83 participants who didn’t know their serostatus (either never been tested or results unknown). Tables 1, 2 and 3 show the comparisons of socio-demographic and drug consumption characteristics of self-reported HIV, HCV and co-infection.

**Table 1:** Socio-demographic and drug consumption variables associated to self-reported HIV, from drug users in Catalonia, 2012

	Total	HIV positive	HIV negative	Bivariate analysis^a^	Multivariate analysis^b^
	*n* = 512	*n* = 76 (%)	*n* = 436 (%)	PR (95 % CI)	PR (95 % CI)
Age, median (IQR)	39 [35–44]	41 [37–45]	36 [32–43]	1 (1.02–1.06)	1.007 (0.98–1.04)
Sex					
Men	392 (76.56)	54 (13.78)	338 (86.22)	1	1
Women	120 (23.44)	22 (18.33)	98 (81.67)	1.32 (0.84–2.08)	1.96 (1.27–3.04)
Country of birth					
Spain	462 (90.23)	67 (14.50)	395 (85.50)	1	
Other countries	48 (9.38)	8 (16.67)	40 (83.33)	1.15 (0.59–2.26)	
Unknown	2 (0.39)	1 (50)	1 (50)	–	
Current marital status					
Single	275 (53.71)	46 (16.73)	229 (83.27)	1	
Married	120 (23.44)	18 (15)	102 (85)	0.89 (0.54–1.47)	
Divorced	89 (17.38)	12 (13.48)	77 (86.52)	0.80 (0.44–1.44)	
Unknown	28 (5.47)	–	28 (100)	–	
Educational level					
Primary education or less	155 (30.27)	28 (18.06)	127 (81.94)	1	
Secondary education	203 (39.65)	27 (13.30)	176 (86.70)	0.73 (0.45–1.19)	
Undergraduate education	154 (30.08)	21 (13.64)	133 (86.36)	0.75 (0.45–1.26)	
Current employment status					
Current regular job	111 (21.68)	2 (1.80)	109 (98.20)	1	1
Unemployment	277 (54.1)	38 (13.72)	239 (86.28)	7.75 (1.9–31.59)	5.00 (1.26–19.91)
Disabled or retired	99 (19.34)	35 (35.35)	64 (64.65)	19.98 (4.93–80.94)	8.92 (2.24–35.60)
Others, including never has worked and worked at home	25 (4.88)	1 (4)	24 (96)	2.26 (0.21–23.96)	1.71 (0.22–12.99)
Number of years by injecting drug consumption					
Non-injecting users	286 (55.86)	8 (2.80)	278 (97.20)	1	1
Injection for <6 years	58 (11.33)	10 (17.24)	48 (82.76)	6.19 (2.55–15.00)	4.88 (2.08–11.46)
Injection for 6 to 12 years	27 (5.27)	7 (25.93)	20 (74.07)	9.30 (3.65–23.68)	7.73 (3.08–19.37)
Injection 13 to 22 years	68 (13.28)	29 (42.65)	39 (57.35)	15.30 (7.32–31.96)	12.10 (5.76–25.41)
Injection for >22 years	52 (10.16)	18 (34.62)	34 (65.38)	12.42 (5.70–27.05)	7.73 (3.08–19.37)
Injection for unknown time	21 (4.1)	4 (19.05)	17 (80.95)	6.52 (2.13–19.98)	4.30 (1.19–15.52)
Years after starting drug use, median (IQR)	8.5 [2.4–13.77]	17 [4.80–22.75]	0 [0–4.8]	1.05 (1.03–1.08)	
Period of onset of the 1^st^ drug use					
2001–2012	51 (9.96)	3 (5.88)	48 (94.12)	1	
1991–2000	199 (38.87)	15 (7.54)	184 (92.46)	1.28 (0.38–4.24)	
1981–1990	195 (38.09)	44 (22.56)	151 (77.44)	3.82 (1.24–11.79)	
1953–1980	64 (12.5)	13 (20.31)	51 (79.69)	3.45 (1.04–11.47)	
Unknown period	3 (0.59)	1 (33.33)	2 (66.67)	5.67 (0.81–39.46)	
Heroin use					
No	216 (42.19)	5 (2.31)	211 (97.69)	1	
Yes	296 (57.81)	71 (23.99)	225 (76.01)	10.46 (4.30–25.46)	
Prison History					
No	393 (76.56)	45 (11.45)	348 (88.55)	1	
Yes	119 (23.24)	31 (26.05)	88 (73.95)	3.52 (1.95–6.37)	

*HIV* human immunodeficiency virus, *IQR* interquartile range, *PR* prevalence ratio, *CI* confidence intervals

^a^Univariate Poisson regression models with robust variance

^b^Multivariate Poisson regression model included covariates determined to be statistically important on the basis of associations with infection in the univariate analysis (*p* < 0.2)

**Table 2:** Socio-demographic and drug consumption variables associated to self-reported HCV, from drug users in Catalonia, 2012

	HCV positive	HCV negative	Bivariate analysis^a^	Multivariate analysis^b^
	*n* = 203 (%)	*n* = 309 (%)	PR (95 % CI)	PR (95 % CI)
Age, median (IQR)	42 [37–46]	35 [31–41]	1.05 (1.04–1.06)	1.02 (1.01–1.03)
Sex				
Men	159 (40.56)	233 (59.44)	1	1
Women	44 (36.67)	76 (63.33)	0.9 (0.69–1.17)	1.15 (0.99–1.36)
Country of birth				
Spain	179 (38.74)	283 (61.26)	1	
Other countries	22 (45.83)	26 (54.17)	1.19 (0.86–1.65)	
Unknown	2 (100)	–		
Current marital status				
Single	112 (40.73)	163 (59.27)	1	
Married	51 (42.50)	69 (57.50)	1.04 (0.8–1.33)	
Divorced	33 (37.08)	56 (62.92)	0.9 (0.66–1.22)	
Unknown	7 (25)	21 (75)	–	
Educational level				
Primary education or less	71 (45.81)	84 (54.19)	1	
Secondary education	80 (39.41)	123 (60.59)	0.86 (0.67–1.09)	
Undergraduate education	52 (33.77)	102 (66.23)	0.73 (0.55–0.97)	
Current employment status				
Current regular job	17 (15.32)	94 (84.68)	1	1
Unemployment	109 (39.35)	168 (60.65)	2.62 (1.65–4.15)	1.52 (1.15–2.56)
Disabled or retired	69 (69.70)	30 (30.30)	4.63 (2.93–7.32)	1.77 (1.30–2.40)
Others, including never has worked and worked at home	8 (32.0)	17 (68.0)	2.13 (1.04–4.37)	1.51 (1.12–2.06)
Number of years by injecting drug consumption				
Non–injecting users	16 (5.59)	270 (94.41)	1	1
Injection for <6 years	35 (60.34)	23 (39.66)	10.82 (6.44–18.2)	5.88 (3.27–13.74)
Injection for 6 to 12 years	23 (85.19)	4 (14.81)	15.28 (9.25–25.23)	7.88 (4.46–13.94)
Injection 13 to 22 years	63 (92.65)	5 (7.35)	16.62 (10.28–26.88)	7.77 (4.40–13.74)
Injection for >22 years	52 (100)	–	17.94 (11.14–28.88)	7.98 (4.49–14.19)
Injection for unknown time	14 (66.67)	7 (33.33)	11.42 (6.45–20.21)	5.89 (3.14–11.03)
Years after starting drug use, median (IQR)	26 [20–30]	17 [12–23]	1.07 (1.05–1.08)	5.80 (3.09–10.91)
Period of onset of the 1^st^ drug use				
2001–2012	2 (3.92)	49 (96.08)	1	
1991–2000	46 (23.12)	153 (76.88)	5.87 (1.47–23.36)	
1981–1990	111 (56.92)	84 (43.08)	14.44 (3.69–56.49)	
1953–1980	43 (67.19)	21 (32.81)	17.13 (4.36–67.37)	
Unknown period	1 (33.33)	2 (66.67)	8.5 (1.04–69.35)	
Heroin use				
No	10 (4.63)	206 (95.37)	1	1
Yes	193 (65.20)	103 (34.80)	14.21 (7.72–26.19)	2.86 (1.42–5.75)
Prison history				
No	124 (31.55)	269 (68.45)	1	
Yes	79 (66.39)	40 (33.61)	3.35 (2.41–4.65)	

*HCV* Hepatitis C virus, *IQR* interquartile range, *PR* prevalence ratio, *CI* confidence intervals

^a^Univariate Poisson regression models with robust variance

^b^Multivariate Poisson regression model included covariates determined to be statistically important on the basis of associations with reported infection in the univariate analysis (*p* < 0.2)

**Table 3:** Socio-demographic and drug consumption variables associated to self-reported co-infection (HIV/HCV). Drug users in Catalonia, 2012

	Positive Co-infection HIV/HCV	Negative Co-infection HIV/HCV	Bivariate analysis^a^	Multivariate analysis^b^
	*n* = 69 (%)	*n* = 443 (%)	PR (95 % CI)	PR (95 % CI)
Age, median (IQR)	42 [37–45]	37 [32–43]	1.05 (1.03–1.07)	1.01 (0.99–1.04)
Sex				
Men	52 (13.27)	340 (86.73)	1	1
Women	17 (14.17)	103 (85.83)	1.06 (0.64–1.77)	1.62 (1.01–2.60)
Country of birth				
Spain	61 (13.20)	401 (86.80)	1	
Other countries	7 (14.58)	41 (85.42)	1.11 (0.54–2.29)	
Unknown	1 (50)	1 (50)		
Current marital status				
Single	42 (15.27)	233 (84.73)	1	
Married	18 (15)	102 (85)	0.97 (0.59–1.62)	
Divorced	9 (10.11)	80 (89.89)	0.66 (0.33–1.29)	
Unknown	–	28 (100)	–	
Educational level				
Primary education or less	24 (15.48)	131 (84.52)	1	
Secondary education	25 (12.32)	178 (87.68)	0.79 (0.47–1.33)	
Undergraduate education	20 (12.99)	134 (87.01)	0.52 (0.83–0.48)	
Current employment status				
Current regular job	1 (0.90)	110 (99.10)	1	1
Unemployment	34 (12.27)	243 (87.73)	13.87 (1.92–100.11)	8.16 (1.17–56.92)
Disabled or retired	33 (33.33)	66 (66.67)	37.67 (5.25–270.38)	14.10 (1.98–100.48)
Others, including never has worked and worked at home	1 (4)	24 (96)	4.52 (0.29–69.84)	3.24 (0.28–37.89)
Number of years by injecting drug consumption				
Non-injecting users	3 (1.05)	283 (98.95)	1	1
Injection for <6 years	10 (17.24)	48 (82.76)	16.49 (4.68–58.09)	13.04 (3.80–44.78)
Injection for 6 to 12 years	7 (25.93)	20 (74.07)	24.80 (6.80–90.43)	19.50 (5.39–70.55)
Injection 13 to 22 years	28 (41.18)	40 (58.82)	39.39 (12.34–125.78)	29.07 (9.06–93.24)
Injection for >22 years	18 (34.62)	34 (65.38)	33.12 (10.12–108.42)	22.77 (6.90–75.19)
Injection for unknown time	3 (14.29)	18 (85.71)	13.05 (2.80–60.88)	6.98 (1.25–39.06)
Years after starting drug use, median (IQR)	26 [21–30]	20 [14–27]	1.06 (1.04–1.09)	
Period of onset of the 1^st^ drug use				
2001–2012	1 (1.96)	50 (98.04)	1	
1991–2000	12 (6.03)	187 (93.97)	3.06 (0.41–22.99)	
1981–1990	42 (21.54)	153 (78.46)	10.93 (1.54–77.51)	
1953–1980	13 (20.31)	51 (79.69)	10.36 (1.40–76.58)	
Unknown period	1 (33.33)	2 (66.67)	17.00 (1.37–210.31)	
Heroin use				
No	2 (0.93)	214 (99.07)	1	
Yes	67 (22.64)	229 (77.36)	24.67 (6.11–99.60)	
Prison history				
No	39 (9.92)	354 (90.08)	1	
Yes	30 (25.21)	89 (74.79)	3.90 (2.04–7.45)	

*HCV* Hepatitis C virus, *HIV* human immunodeficiency virus, *IQR* interquartile range, *PR* prevalence ratio, *CI* confidence intervals

^a^Univariate Poisson regression models with robust variance

^b^Multivariate Poisson regression model included covariates determined to be statistically important on the basis of associations with co-infection in the univariate analysis (*p* < 0.2)

The three self-reported serological status groups shared similar associations. The median age of self-reported infected subjects was 41 years for HIV and 42 years for HCV and HIV/HCV co-infection. The prevalence of self-reported HIV was 18.33 % among women and 13.78 % among men (*p* = <0.001). Among self-reported HCV cases, a higher proportion was found among men (40.56 %), compared to women (36.67 %), whereas for self-reported co-infection cases, the proportion was slightly higher for women (14.17 % vs 13.27 % for men).

The prevalence of HIV, HCV and co-infection increased with early onset of drugs consumption. In the univariate analysis for the three self-reported serostatus scenarios, long periods of drug injection were found to be associated to self-reported infection (participants with longer periods of injection being more likely to self-report infection). Self-reported infections among participants who started drug consumption more recently (2001–2012), [51(9.96 %)] were much less common, with only a few cases of HIV [3(3.95 %)], HCV [2(0.99 %)] and co-infection [1(1.45 %)]. In the multivariate analysis heroin use was only found to be significant for self-reported HCV infection. Nevertheless, 10 HCV positive participants did not report heroin consumption, just cocaine.

Table 4 describes self-reported serostatus according to informed parenteral use ever. From the 510 subjects with known history of parenteral use, 224 (43.92 %) individuals reported having injected drugs at least once, and of these, 187 (83.48 %) individuals self-reported to be positive for HCV and 68 (30.36 %) for HIV, 66 (29.46 %) individuals were co-infected. Among the drug users who reported never having injected drugs (*N* = 286, 56.08 %), 164 (57.34 %) participants self-reported being negative for both infections the last time they were tested, and 101 (35.44 %) were unaware of their serostatus either for one infection or both. Twenty-one (7.34 %) participants self-reported a positive serostatus for HIV and/or HCV but did not report any parenteral use. Among these 21 individuals, 16 self-reported being positive for HCV, and eight for HIV, three reported co-infection. Only two people declared exchanging sex for money. For those that reported a negative test, the median time since the last HIV test was 11.41 months (inter-quartile range (IQR) 4-12) and for the HCV test was 4.5 months (IQR 2–7).

**Table 4:** Self-reported HCV and/or HIV positive, negative and unknown serostatus by parenteral use^a^. Catalonia, 2012

	Injecting drug users	Non-injecting users	Total	*p*-value^b^
	*n* = 224 (%)^c^	*n* = 286 (%)^d^	*n* = 510^f^ (%)^e^	
HCV	187 (83.48)	16 (5.59)	203 (39.80)	<0.001
HIV	68 (30.36)	8 (2.80)	76 (14.90)	<0.001
HCV + HIV	66 (29.46)	3 (1.05)	69 (13.53)	<0.001
Negative self-report of HCV and HIV	26 (11.61)	164 (57.34)	190 (37.25)	
Unknown serostatus for HCV	3 (1.34)	24 (8.39)	27 (5.29)	
Unknown serostatus for HIV	4 (1.79)	23 (8.04)	27 (5.29)	
Unknown serostatus for HCV and HIV	2 (0.89)	54 (18.88)	56 (10.98)	

*HIV* human immunodeficiency virus, *HCV* Hepatitis C virus

^a^First three rows in the table are not mutually exclusive

^b^Chi square test

^c^Percentages are the proportion among injecting drug users (IDUs). They do not add up to 100 because co-infected individual count in several categories

^d^Percentages are the proportion among drug users who have never injected drugs. They do not add up to 100 because co-infected individual count in several categories

^e^Percentages are the proportion among the total. They do not add up to 100 because co-infected individual count in several categories

^f^Two cases were not included into the analysis due to missing information related with history of drug injection. One reported negative serostatus and the other one unknown HIV serostatus

## Discussion

In this study of drug users in Catalonia, a high prevalence of self-reported HCV (39.65 %) was observed, with moderate prevalence of self-reported HIV (14.84 %) and co-infection (13.48 %). As expected these self-reported infections were strongly associated with parenteral drug use and length of drug consumption. However, women were more likely to report being HIV infected or co-infected, and there was a high proportion of drug users who have never injected drugs who were unaware of their serological status. A small proportion (7.34 %) of drug users reporting never having injected drugs reported being infected.

These results are in line with those of other studies which suggest that despite the introduction of preventive interventions (screening, counselling, needle and syringe exchange programs, methadone programs and other treatments for substance abuse), transmission persists and prevalence of blood borne infections, particularly HCV, continues to be notable in high-risk groups [16], such as drug users especially IDUs. Currently in Spain, the route of HIV transmission for 28 % of all Acquired Immunodeficiency syndrome (AIDS) cases is IDU [17], and in Europe and the USA, IDU’s account for more than 60 % of new HCV infections [4, 17].

Our results showed that women who use drugs had a higher likelihood to self-report HIV infection, than men who use drugs, but unfortunately, sexual transmission factors were not collected. Some studies have reported that women who use drugs more often engage in risky sexual behaviours, being more likely than men to have unprotected sex, to have multiple sex partners, to engage in sex for money or drugs, to use a greater variety of drugs than men and to be at a higher risk of suffering violence and marginalization [18–21]. Despite a decrease in the HIV/AIDS epidemic among IDUs, significant gender differences persist. Women in drug care facilities constitute a minority, and their gender specific needs may often be overlooked. Therefore, in the implementation of HIV prevention strategies, it is crucial to consider a gender perspective when focusing needs of drug consumers.

Comparing our study with others conducted in Catalonia [15, 16], prevalence of BBI are similar. However, we have found a large number of individuals with unknown serostatus, especially among drug users who have never injected drugs. In the literature, researchers and policymakers have a tendency to focus BBI intervention on IDUs, ignoring the risk of individuals who use drugs by other routes. Although one of the most important predictors for BBI seroconversion is starting injection [22], many risks have been described among drug users who have never injected drugs, such as high levels of mixing or “bridging” with the IDU population [23], and high levels of unprotected sex [24, 25]. Since 2008 the Catalan Surveillance system for HIV and HCV (in persons who inject drugs) recruits from harm reduction centres IDUs having injected drugs in the previous 6 months. There is no formal behavioural surveillance system for drug users who don’t inject. Therefore behavioural surveillance should regularly monitor rates of transmission from IDUs to drug users who have never injected drugs, by including non-injecting users in the surveillance system, in order to enable the implementation of timely interventions. In fact, the literature describes that behavioural surveillance systems should be integrated in prevention programs that reduce the likelihood of switching from non-injecting routes to drug injection [26–28]. Also, it is important to offer periodic/follow-up and testing access to persons with ongoing risk factors for exposure to BBIs, and to offer information to infected persons; particularly to those who use drugs by injection, about precautions to avoid exposing others to infected blood.

## Strengths and limitations

One of the strengths of this study is the large sample size and the fact that data were collected from different types of centres and in towns of different sizes in Catalonia, thus contributing to the study’s external validity. Results might reflect changes in the epidemiology of HIV and HCV in drug users seeking treatment for substance abuse or safe injecting places in urban areas. However, as the sample was recruited only from healthcare centres, we cannot extrapolate our results to drug users who do not access these centres. Nevertheless, our results do not differ greatly from those of studies with street-recruited participants (i.e. not selected from therapeutic centres) [13].

Among other limitations of this paper we must mention, first, that it is not possible to make causal inferences due to the study design. However in order to achieve a more precise assessment of the association in this type of study design, we used a Poisson regression model. Second, the rate of BBI positivity may be an underestimate, first because there was a high proportion of individuals who reported not having had a serological test in the last six months and second because it was based on self-reported serostatus, and thus subject to recall and social desirability biases. In order to reduce the latter, we stressed to participants that their anonymity was guaranteed. Besides, different studies have shown that the main results in cross sectional studies are not seriously affected by these forms of bias, and that the results obtained may be valid despite being self-reported [29]. Third, it is important to mention that it was not possible to confirm serological status among positive cases, and that among negative cases there is still quite a high chance that they could be infected without knowing it. Even so, prevalence of infection obtained in this study does not differ greatly compared to other studies among IDUs [15] .

## Conclusions

Among drug users in Catalonia, prevalence of HIV, HCV and co-infection is still a big issue, especially among IDUs. However, women and drug users who have never injected drugs are groups with a significant risk of infection; this might be related to their engaging in high-risk behaviours and to ignorance of their serological status. Therefore, it is crucial to strengthen behavioural surveillance systems in order to ascertain the serostatus of target populations such as drug users who have never injected drugs, offering rapid testing, ensuring access to health care and planning preventive strategies adapted to the profile of specific subpopulations with a gender-sensitive approach.
